# DNA Methylation in macrophages infected with *Leishmania* spp. in different culture conditions

**DOI:** 10.1080/22221751.2025.2508766

**Published:** 2025-05-21

**Authors:** Eleonora Loi, Paola Andrea Barroso, Agustín Moya Alvarez, Patrizia Zavattari, Ana Florencia Vega Benedetti

**Affiliations:** a Department of Biomedical Sciences, Unit of Biology and Genetics, University of Cagliari, Cagliari, Italy; b Institute of Experimental Pathology Dr. Miguel Ángel Basombrío - CONICET, University of Salta, Salta, Argentina

**Keywords:** DNA methylation alterations, CpG islands, *Leishmania* infection, *Leishmania*-host cell interaction, *Leishmania*-associated alterations, Glucantime, Amphotericin B

## Abstract

*Leishmania* modulate the host cell epigenome, including DNA methylation. This work aimed to explore the DNA methylation pattern in infected macrophages with *L. (Viannia) braziliensis*, *L. (Leishmania) amazonensis* and *L. (L.) infantum*. We performed a genome-wide methylation analysis in macrophages, cultured in presence/absence of interleukin 6 (IL-6) and infected with the three species for a period of 72hs. Upon 72hs infection, sample groups of *L. (V.) braziliensis* – and *L. (L.) infantum*-infected macrophages were treated with Glucantime and Amphotericin B for 48hs, respectively. Uninfected macrophages and macrophages treated with heat-killed *Leishmania* were included as controls. Several CpG islands alterations were identified upon infection and among species. The methylome analysis showed that *L. (L.) amazonensis* and *L. (L.) infantum* clustered together separately from *L. (V.) braziliensis*. The identified alterations were mainly associated with cytoskeleton organization. We also detected that the DNA methylation pattern of ten, six and eight CGIs for each aforementioned species slightly changed in a culture environment with IL-6, whereas treatment led to distinct DNA methylation profiles respect to untreated samples. Interestingly, some altered CGIs showed a re-establishment towards the control methylation pattern in *L. (L.) infantum* (69%, 11 out of 16) and *L. (V.) braziliensis* (36%, 4 out of 11). The identified alterations suggest a species-specific parasite/host interaction probably leading to gene expression regulation. The discovery of these methylation alterations addresses further functional studies and suggests them as potential therapeutic targets.

## GRAPHICAL ABSTRACT

**Figure:**
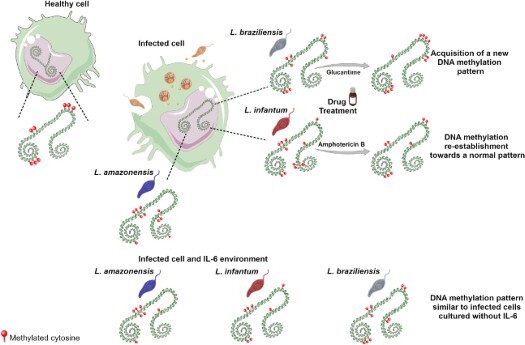

## Introduction

Leishmaniasis is a major neglected tropical disease caused by protist species of *Leishmania* genus that presents a broad diversity of clinical forms [1]. In north Argentina species responsible of cutaneous/muco-cutaneous forms include *L. (Viannia) braziliensis*, *L. (V.) guyanensis* and *L. (Leishmania) amazonensis*, while visceral leishmaniasis can be caused by *L. (L.) infantum* [2]. Although improvements in diagnosis and intervention strategies have been achieved, there are not effective vaccines or therapies. Current drug therapies such as antimonials (Glucantime) and Amphotericin B, recommended by the World Health Organization, lead to adverse effects, drug resistance or treatment failure [3]. The mechanism of action of Glucantime is still unclear but it has been proposed that the pentavalent antimony is reduced to the trivalent form responsible for parasite killing [4]. Previous works demonstrated that this drug induces DNA damage mediated by oxidative stress supporting its mutagenic effect [5]. Instead, Amphotericin B exerts its effect by binding to ergosterol-related sterols, abundant in the *Leishmania* membrane, resulting in the formation of pores and consequent cell death [3].

*Leishmania* have a dixenous life cycle, alternating two stages. The flagellated promastigote transforms into the non-motile amastigote in host macrophages where the protists multiply. Pathogens including *Leishmania* evolved mechanisms to avoid macrophage parasiticidal action, transforming the phagolysosome in their safe niche [6]. One strategy to elude host response is the modulation of the host epigenome, including DNA methylation, histone modifications and non-coding RNAs [7]. This is an emerging area in the investigation of parasite infections and achieves relevance in host/parasite interactions. DNA methylation, a reversible signature, contributes to gene expression regulation and maintains genome stability. It mainly occurs at CpG sites throughout the genome, in regulatory regions, at DNA repetitive elements and gene bodies. CpG-enriched regions, defined as CpG islands (CGIs), are often located at promoters [7]. Marr *et al*. reported several differentially methylated CpG sites in macrophages upon *L. (L.) donovani* infection [8]. Moreover, low methylation level at *FLI1* promoter was observed in cutaneous lesions from patients and a downward trend in its methylation level in interleukin 6 (IL-6) treated macrophages infected by *L. (V.) braziliensis* [9]. IL-6, secreted by several immune cells, presents a controversial role in leishmaniasis acting as anti-inflammatory/pro-inflammatory cytokine [10]. *In vivo* experiments have shown that the absence of IL-6 leads to parasite killing supporting its immunopathological role whereas other evidence reported opposite effects suggesting a host-protective function [10–13]. This dual nature of IL-6 in leishmaniasis in the context of DNA methylation has not been deeply considered.

Given the heterogeneity in macrophage protein expression and in the immune response among clinical forms, it is probable that each *Leishmania* species differently modifies the host cell epigenome [14,15]. The focus on the modifications of the host DNA methylome may provide new druggable targets and a complement in the diagnostic strategy.

Therefore, in the present study a genome-wide methylation analysis was performed in macrophages infected with *L. (L.) infantum*, *L. (V.) braziliensis* and *L. (L.) amazonensis*. The experiments were performed in the presence/absence of IL-6 to elucidate its possible impact on DNA methylation. Another condition included Glucantime and Amphotericin B treatment in *L. (V.) braziliensis*/*L. (L.) infantum*-infected macrophages, respectively.

## Materials and methods

### Cell culture

U937 cells, obtained from IMBICE (Buenos Aires, Argentina), were cultured in RPMI-1640 containing 10% (v/v) heat inactivated fetal bovine serum (FBS), 100 U/ml penicillin and 50 μg/ml streptomycin (Pen/Strep) at 37°C, 5% CO_2_.

### *Leishmania* culture

Promastigotes of *L. (V.) braziliensis* (MHOM/BR/75/M2903), *L. (L.) amazonensis* (MHOM/BR/1973/M2269) and *L. (L.) infantum* (MHOM/MA/67/ITMAP-263) were cultured during 4 days in Difco blood agar (USMARU) medium with 20% defibrinated rabbit blood and sterile proline balanced salts solution (PBSS) containing 100 U/ml and 50 μg/ml Pen/Strep at 23°C [16]. Then, in order to infect macrophages, the parasites were passed to RPMI 1640 medium supplemented with 10% (v/v) FBS and Pen/Strepduring 6 days.

### Macrophage infection with *Leishmania* species

**Figure 1.**
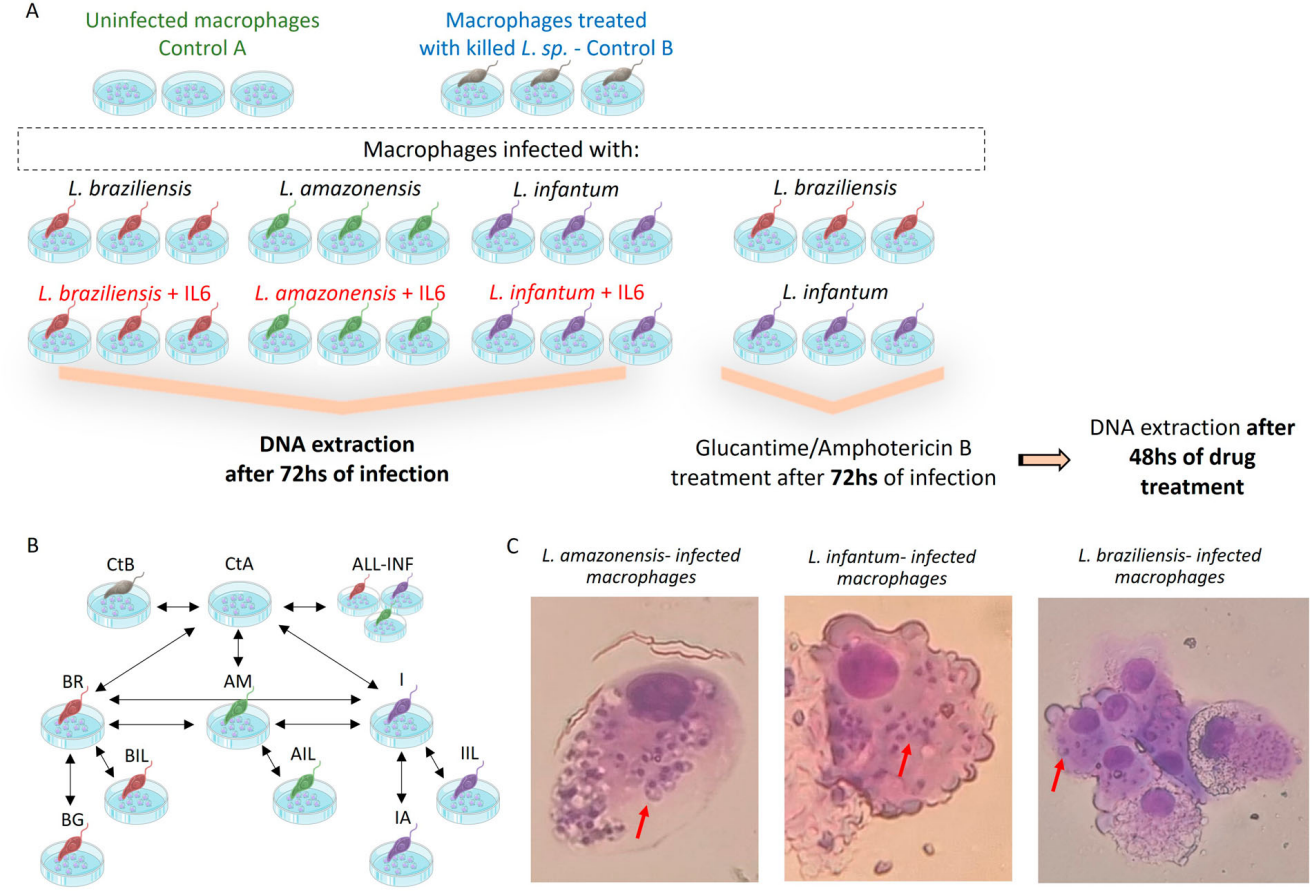
Experimental plan, DNA methylation analysis workflow and infection efficacy. (A) The control groups included uninfected macrophages and macrophages treated with heat-killed *Leishmania*. Macrophages were cultured in the absence or presence of IL-6 and infected with *L. (V.) braziliensis*, *L. (L.) amazonensis* and *L. (L.) infantum*. At 72hs post-infection cells were harvested for DNA extraction. Simultaneously, macrophages infected with *L. (V.) braziliensis* and *L. (L.) infantum* were treated during 48hs with Glucantime and Amphotericin B, respectively. Figure created using Servier Medical Art images. (B) Scheme of differential methylation analyses among conditions. The arrows indicate the paired comparisons. Abbreviations: control A, uninfected macrophages (CtA) and control B, macrophages with heat-killed *Leishmania* (CtB); all infected samples (ALL-INF); *L. (L.) amazonensis*-, *L. (L.) infantum* – and *L. (V.) braziliensis* – infected samples in absence and presence of IL-6 (AM and AIL; I and IIL; BR and BIL); treated *L. (L.) infantum* and *L. (V.) braziliensis* samples (IA and BG). (C) Macrophages infected with *L. (L.) amazonensis*, *L. (L.) infantum* and *L. (V.) braziliensis* amastigotes. Arrows indicate the amastigotes.

The procedure of sample infection and collection is described in Figure 1A.

U937 cells (8 × 10^5^ cells/ml) were seeded in flasks in different conditions in triplicate. Cells were differentiated with phorbol 12-myristate 13-acetate (10 ng/ml) per 48hs at 37°C, constant humidity and 5% CO_2_. The controls included uninfected macrophages and macrophages treated with heat-killed *Leishmania* (65°C for 45 min) [8]. The infected samples were cultured in the absence/presence of IL-6 (20 ng/ml), 24hs before infection according to Almeida *et al*. [9]. Macrophages were infected overnight with promastigotes of *L. (V.) braziliensis*, *L. (L.) amazonensis* and *L. (L.) infantum* at a ratio of 10 parasites per macrophage. Cells infected with *L. (V.) braziliensis* and *L. (L.) amazonensis* were incubated at 33°C, 5% CO_2_, while cells infected with *L. (L.) infantum* were incubated at 37°C, 5% CO_2_ incubator. Remaining extracellular parasites were removed by washing the cultures with PBSS. Samples were further incubated for 72hs to allow promastigotes transform into amastigotes. Afterwards cells were harvested for DNA extraction. Simultaneously, macrophages infected with *L. (V.) braziliensis* and *L. (L.) infantum* were treated per 48hs with Glucantime (80 μg/ml) and Amphotericin B (0.0001 μg/ml), respectively. Of note, the subgenus and first line therapy are different between *L. braziliensis* (*Viannia* and Glucantime treatment) and *L. infantum* and *L. amazonensis* (*Leishmania*, treated with Amphotericin B and Glucantime respectively), so we decided to select one species for each subgenus and treatment. The doses of Glucantime and Amphotericin B selected for this assay were below the cytotoxic dose reported by Sosa *et al*. and Sesana *et al*. [17,18]. Infection rates (percentage of infected macrophage) were determined under a light optical microscope examining 100 cells stained with Diff Quick (Biopack).

### DNA extraction

DNA was extracted from three independent assays of each condition using TRIzol. DNA quantity and purity were assessed by spectrophotometric and fluorometric readings.

### Whole genome methylation study

DNA was bisulfite-converted using EZ DNA Methylation Gold Kit (Zymo Research) following the manufacturer’s instruction. Genome-wide methylation analysis was performed by Illumina Infinium MethylationEPIC BeadChips according to the protocol guide. EPIC BeadChips allow to analyse over 850,000 methylation sites per sample at single-nucleotide resolution. The methylation score for each CpG site is represented as β values according to the fluorescent intensity ratio between methylated probe intensity and the overall intensity. β values range from 0 (unmethylated) to 1 (completely methylated).

### DNA methylation analysis

Raw methylation data were analysed using RnBeads [19]. A differential methylation analysis at CGIs was performed in the following groups (Figure 1B):

- Controls: uninfected macrophages vs heat-killed *Leishmania* infected macrophages. The identified altered CGIs were removed from the other analyses to avoid alterations associated with phagocytosis.
- All *Leishmania* infected vs uninfected macrophages.
- *Leishmania* species-specific infected: *L. (L.) amazonensis*-infected samples /*L. (L.) infantum*-infected samples /*L. (V.) braziliensis*-infected samples vs uninfected macrophages.
- Among *Leishmania* spp. infected samples: *L. (L.) amazonensis* vs *L. (L.) infantum*, *L. (L.) amazonensis* vs *L. (V.) braziliensis* and *L. (V.) braziliensis* vs *L. (L.) infantum*.
- *Leishmania* species-specific infected samples in presence of IL-6 vs in absence of IL-6: *L. (L.) amazonensis* + IL-6 vs – IL-6; *L. (L.) infantum* + IL-6 vs – IL-6 (*I*); *L. (V.) braziliensis* + IL-6 vs – IL-6.
- Drug treated vs infected untreated samples: untreated vs Amphotericin B-treated *L. (L.) infantum*; untreated vs Glucantime-treated *L. (V.) braziliensis*.

CGI annotation was performed according to Illumina Manifest and associated with the nearest genes and transcripts using R annotation package FDb.InfiniumMethylation.hg19 [20]. We selected differentially methylated CGIs (|Δβ| > 0.1, i.e. 10% differential methylation). Methylation alterations were considered significant using *p*-value < 0.05, adjusted or nominal in paired comparisons where the analysis was less robust. Hypermethylation and hypomethylation were defined as Δβ values > 0.1 and Δβ values < −0.1, whereas the threshold was lowered to |Δβ| > 0.05 in the IL-6 and drug treatment analyses since only very few alterations were detected with the stringent threshold. Methylation values of altered CGIs for each sample condition have been used in an analysis of unsupervised hierarchical clustering (UHC) and visualized by Bioconductor package ComplexHeatmap [21].

Further differential methylation analyses between infected samples (*L. (L.) amazonensis*, *L. (L.) infantum* and *L. (V.) braziliensis*) and uninfected macrophages were performed focusing on promoters, defined as the regions 1.5 kb upstream and 0.5 kb downstream of the transcription start sites (TSS) [22].

### Gene enrichment analysis

Functional analyses were carried out using the annotation tool DAVID selecting Reactome pathway [23]. The study was conducted considering all genes associated with the altered CGIs, located at TSS, combining those hyper – and hypo-methylated, identified in *Leishmania* species compared to uninfected macrophages. Since *L. (V.) braziliensis* analysis showed few altered CGIs with |Δβ| > 0.1 we lowered the differential methylation level to |Δβ| > 0.05.

Circos and enrichment plots were generated using the circlize and ggplot R packages [24].

## Results

The infection rates of the three species were: 93% for *L. (L.) amazonensis*, 90% for *L. (L.) infantum* and 72% for *L. (V.) braziliensis* (Figure 1C). In any UHC analyses, biological replicates mostly grouped together excluding any technical procedure bias. Uninfected vs heat-killed *Leishmania* infected macrophages comparison showed only 11 differentially methylated CGIs that were excluded in the subsequent group analyses (Table S1). To note, the heat-killed control was performed using *L. (V.) braziliensis*. As observed in Table S1 some alterations are also significantly altered in some species.

### DNA methylation alterations in macrophages infected by different *Leishmania* species

**Figure 2.**
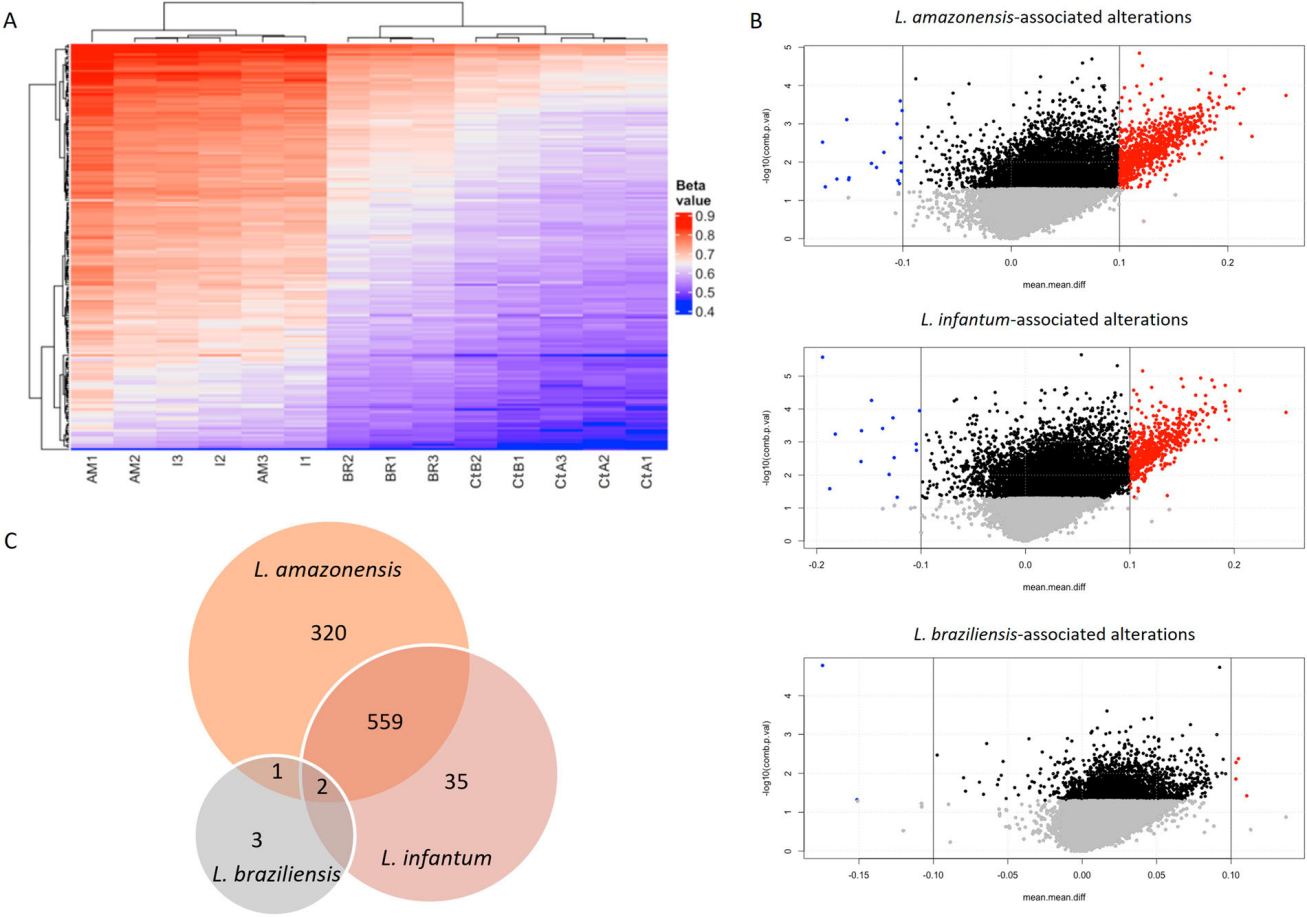
*Leishmania*-associated CGI alterations. (A) Unsupervised hierarchical clustering analysis based on the β value of each *Leishmania* species-infected sample and controls in triplicate. Abbreviations: *L. (L.) amazonensis* (AM1, AM2 and AM3); *L. (L.) infantum* (I1, I2 and I3); *L. (V.) braziliensis* (BR1, BR2 and BR3); uninfected control sample A (CtA1, CtA2 and CtA3) and B, macrophages with heat-killed *Leishmania* (CtB1, CtB2). (B) Volcano plots showing differentially methylated CGIs in *L. (L.) amazonensis*-, *L. (L.) infantum* – and *L. (V.) braziliensis*-infected samples compared to CtA. Significant hypermethylated and hypomethylated CGIs are indicated in red and blue. (C) VennDiagram of the altered CGIs in *Leishmania* species, showing overlapping and species-specific CGIs.

The methylome analysis showed several and different alterations in *L. (L.) amazonensis*, *L. (L.) infantum* and *L. (V.) braziliensis* infected samples vs uninfected macrophages. UHC was performed considering the altered CGIs overlapping TSS between all infected samples and uninfected macrophages (Figure 2A), showing a clear distinction between *L. (L.) amazonensis*/*L. (L.) infantum* and *L. (V.) braziliensis* infected macrophages that clustered with control samples. Comparisons were carried out between each species-specific infection and uninfected macrophages. *L. (L.) amazonensis* showed 865 hypermethylated and 17 hypomethylated CGIs, *L. (L.) infantum* showed 582 hypermethylated and 14 hypomethylated events, whereas few alterations were found in *L. (V.) braziliensis*, four hypermethylated and two hypomethylated CGIs (Figure 2B). We found species-specific alterations and many shared aberrations between *L. (L.) amazonensis* and *L. (L.) infantum* (Figure 2C and Tables S2–S4). The three species share two altered CGIs overlapping with promoter/enhancer regions of *ELOVL6* and *EPB41L4B* encoding for proteins involved in long-chain fatty acids elongation and cytoskeleton structure (Table 1).

**Table 1.** Altered CGIs in common among Leishmania species.

CGI Location	Distance to TSS	Nearest TSS Gene	Δβ *L. braziliensis*/CtA	Δβ *L. infantum*/CtA	Δβ *L. amazonensis*/CtA
chr4:111117463-111118390	1380	*ELOVL6*	0.105	0.179	0.205
chr9:112081403-112082905	115	*EPB41L4B*	0.103	0.115	0.160

Note: uninfected macrophages (CtA: control A), transcription start site (TSS).

The most altered CGIs for each species are reported in Table 2.

**Table 2.** Most altered CGIs for each species.

			*L. infantum* vs CtA		*L. braziliensis* vs CtA		*L. amazonensis* vs CtA	
Location	Distance to TSS	Nearest TSS Gene	Delta beta	comb.*p*.val	Delta beta	comb.*p*. val	Delta beta	comb.*p*.val
chr9:139917179-139917492	19	*ABCA2* (uc004cko.1)	−0.034	0.002	−0.17	1.67E-05	−0.023	0.020
chr7:149469543-149469748	546	*ZNF467* (uc003wgc.3)	0.097	0.018	0.042	0.121	0.174	0.006
chr8:141359156-141359621	57805	*TRAPPC9* (uc010mel.1)	−0.094	0.049	0.034	0.312	−0.174	0.003
chr6:32489743-32490128	7877	*HLA-DRB5* (uc003obj.3)	0.148	1E-04	0.027	0.073	0.07	0.038
chrY:6778575-6780028	0	*TBL1Y* (uc004frb.3)	−0.182	6E-04	−0.055	0.109	−0.066	0.204

Note: uninfected macrophages (CtA: control A), transcription start site (TSS). In red the altered CGIs.

**Figure 3.**
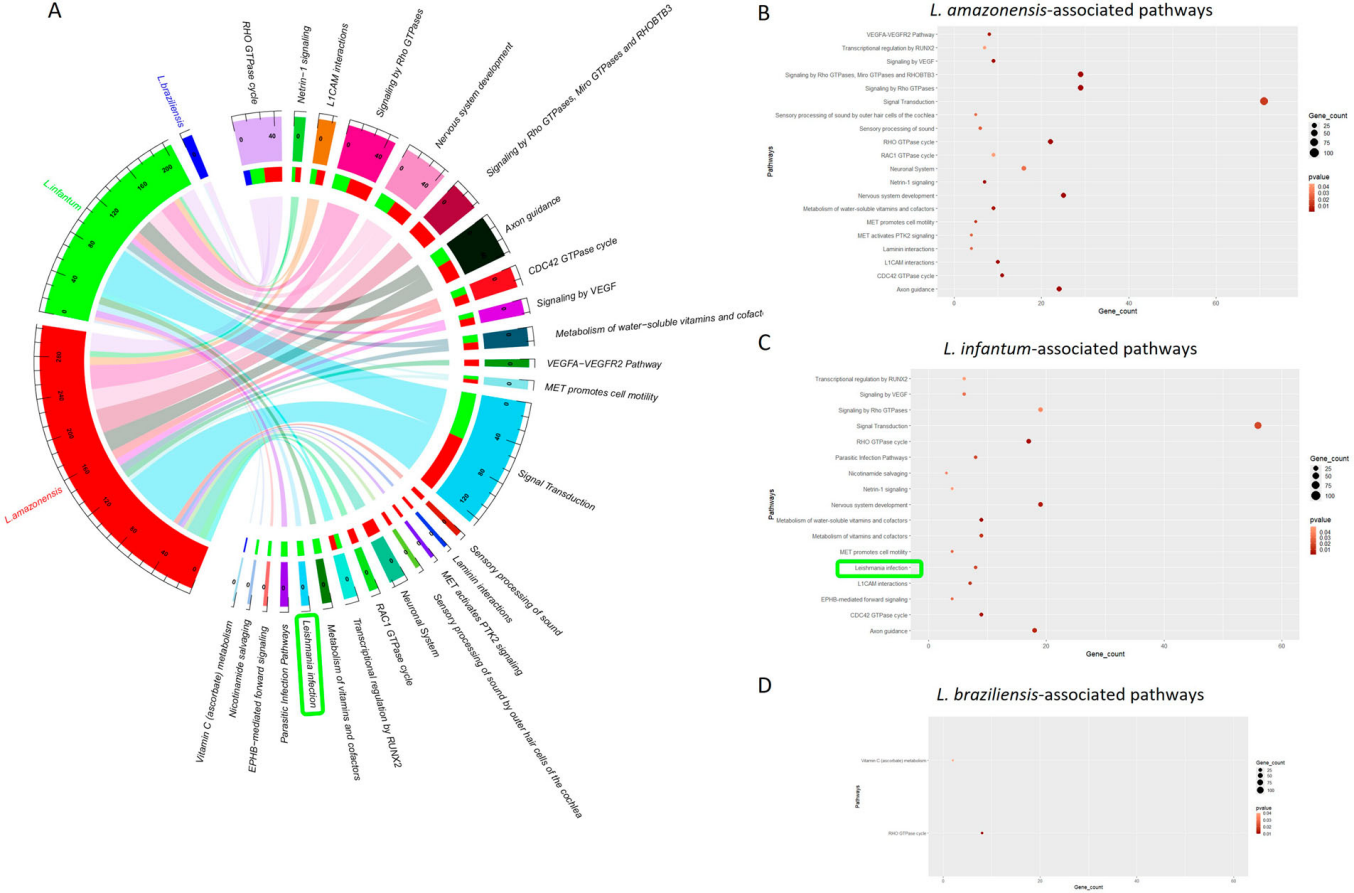
Reactome pathway analysis. (A) Chord diagram showing the significantly altered pathways associated with each *Leishmania* species, including the number of the identified altered genes within the outer ring. The pathways are located on the right outer ring, whereas *L. (L.) amazonensis* (red), *L. (L.) infantum* (green) and *L. (V.) braziliensis* (blue) are on the left outer ring and on the right inner ring overlapping with the aberrant pathways. Colour beams link the pathway with the *Leishmania* species. Enrichment dot plots showing the significant altered pathways with the number of the identified genes in *L. (L.) amazonensis* (B), *L. (L.) infantum* (C) and *L. (V.) braziliensis* (D). The name of the *Leishmania* infection pathways, altered in *L. (L.) infantum* infection, is highlighted in green.

In all species the functional alterations included pathways mainly leading to the dysregulation of cytoskeleton dynamics, cell motility/adhesion/differentiation, and cofactors metabolism (Figure 3A). *L. (L.) amazonensis* was associated with even more proliferative/motility pathways (Figure 3B). *L. (L.) infantum* functional analysis showed *Leishmania* infection pathway (*p*-value = 0.02) whose genes are linked to cell proliferation/adhesion and cytoskeleton organization (Figure 3C). Since many genes are altered in *L. (L.) amazonensis* infection, the *Leishmania* pathway does not reach statistical significance (*p*-value = 0.06). The methylation values of these genes are shown in Table 3. Instead, two pathways were significantly altered in *L. (V.) braziliensis* infection (Figure 3D).

**Table 3.** Altered CGI-associated genes, belonging to Leishmania infection pathway.

		Methylation β values				
CGI location	CGI-associated genes	*L. infantum* infection	*L. braziliensis* infection	*L. amazonensis* infection	CtA	CtB
chr2:204192656-204193740	*ABI2*	0.66	0.58	0.67	0.52	0.55
chr21:27541894-27543524	*APP*	0.68	0.62	0.70	0.57	0.57
chr19:47137781-47138070	*GNG8*	0.70	0.64	0.73	0.58	0.61
chr5:16935556-16936408	*MYO10*	0.64	0.55	0.66	0.51	0.54
chr5:57755335-57756803	*PLK2*	0.69	0.60	0.69	0.56	0.58
chr1:84543147-84544261	*PRKACB*	0.76	0.71	0.77	0.66	0.68
chr20:35974178-35974913	*SRC*	0.78	0.70	0.76	0.66	0.66
chr7:123388400-123389623	*WASL*	0.58	0.52	0.60	0.46	0.48

Note: uninfected macrophages (CtA: control A),; macrophages with heat-killed *Leishmania* (CtB: control B).

### DNA methylation alterations upon *Leishmania* infection in absence/presence of IL-6

The DNA methylation pattern in infected macrophages cultured without IL-6 slightly differed from the ones in an IL-6 environment. In the presence of IL-6, *L. (L.) amazonensis*, *L. (L.) infantum* and *L. (V.) braziliensis* infected samples showed, respectively, ten, six and eight differently methylated CGIs compared to those without IL-6. Almost all CGIs were located far away from TSS, except for three *L. (V.) braziliensis*-associated CGIs: *FAM224B*, *TMEM54* and *NEURL1* (Δβ = 0.12, – 0.05 and – 0.07, respectively).

The UHC study for each species demonstrated that there is not a clear separation between IL-6 conditions (Figure S1).

### DNA methylation alterations in infected macrophages upon drug treatment

**Figure 4.**
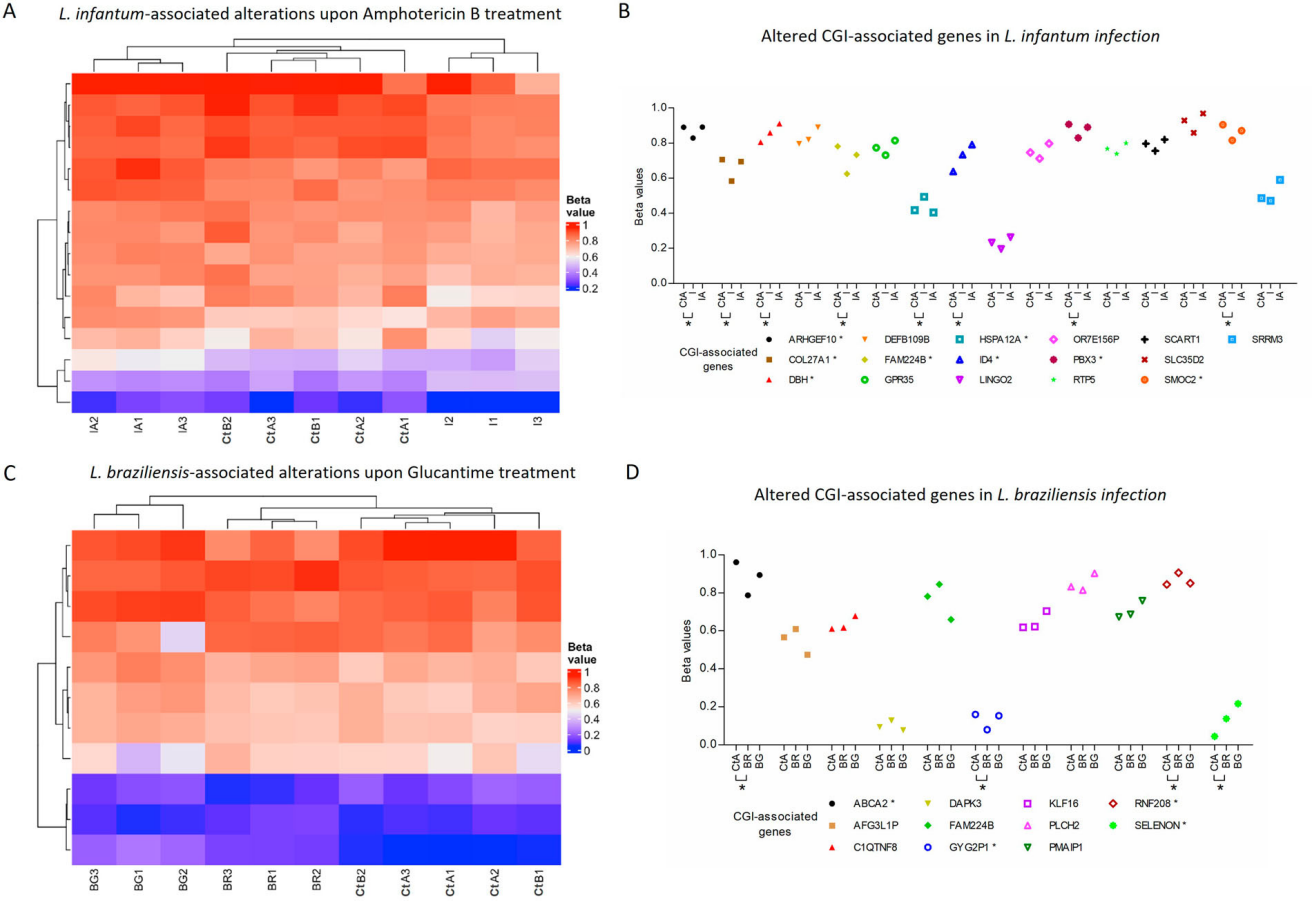
DNA methylation of altered CGIs in treated and untreated macrophages. Heatmaps showing beta methylation values of CGIs in control, treated and untreated samples for *L. (L.) infantum* (A) and *L. (V.) braziliensis* (C) infection in triplicate. Abbreviations: untreated and treated *L. (L.) infantum* (I1, I2 and I3; IA1, IA2 and IA3); untreated and treated *L. (V.) braziliensis* (BR1, BR2 and BR3; BG1, BG2 and BG3); control A, uninfected macrophages (CtA1, CtA2 and CtA3) and B, macrophages with heat-killed *Leishmania* (CtB1, CtB2). Dot plots of mean beta values, resulting from the average of the triplicates for each altered CGI in *L. (L.) infantum* (B) and *L. (V.) braziliensis* (D). Asterisks indicate significant differences between infected and CtA samples (nominal *p* value < 0.05 and |Δβ| > 0.05).

We detected 16 and 11 significantly altered CGIs in *L. (L.) infantum* and *L. (V.) braziliensis* infected samples upon treatment compared to the untreated samples. Amphotericin B treatment in *L. (L.) infantum* infection led to a re-establishment of the DNA methylation level of the CGIs indicated with an asterisk in Figure 4. In fact, the UHC analysis displayed two main branches, one with two clusters including controls and *L. (L.) infantum* treated samples and the second branch with *L. (L.) infantum* untreated macrophages (Figure 4A). The beta values of *L. (L.) infantum* treated samples resemble the ones obtained in controls (Figure 4B). To note, 69% (11 out of 16) of the altered CGIs restore their methylation value towards a control state, while 31% (5 out of 16) acquire a further methylation alteration. Half of the differentially methylated CGIs in *L. (L.) infantum* treated samples compared to the untreated ones, were already significantly altered upon infection, shown with an asterisk in Figure 4B. Instead, *L. (V.) braziliensis* Glucantime-treated macrophages presented further methylation alterations, branching in a separate group (Figure 4C). Few CGIs (36%, 4 out of 11) tend to restore their methylation value upon treatment and four already presented significant differences between uninfected macrophages and *L. (V.) braziliensis* untreated samples, shown with an asterisk in Figure 4D. Interestingly, the CGI associated with *ABCA2*, solely detected in *L. (V.) braziliensis* infection, showed after treatment a methylation value (0.89) closer to uninfected macrophages (0.96) than to the untreated samples (0.78) (Figure 4D).

### DNA methylation at promoters upon *Leishmania* infection

**Figure 5.**
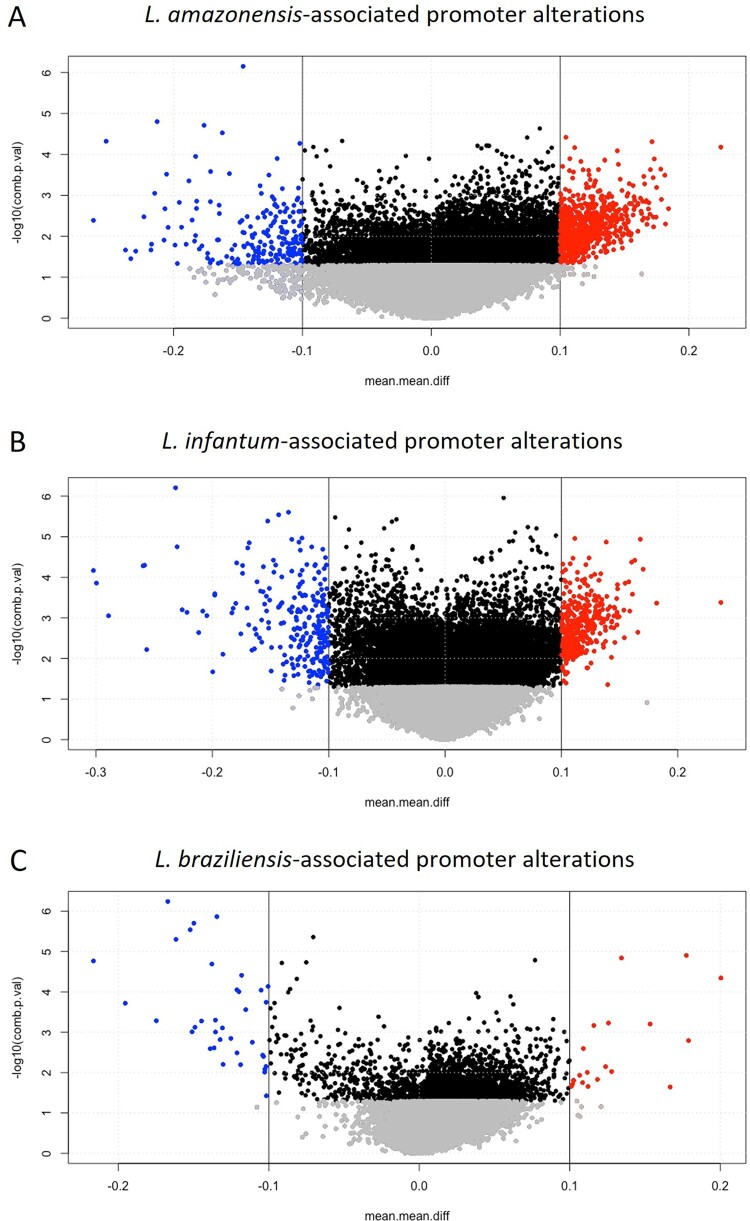
*Leishmania*-associated alterations at promoters. Volcano plots showing differentially methylated promoters in three comparisons: *L. (L.) amazonensis*-infected macrophages vs uninfected macrophages (A), *L. (L.) infantum*-infected macrophages vs uninfected macrophages (B) and *L. (V.) braziliensis*-infected macrophages vs uninfected macrophages (C). Significant hypermethylated and hypomethylated CGIs are indicated in red and blue.

Several hyper-/hypo-methylated promoters were found, 711/210 in *L. (L.) amazonensis*, 361/250 in *L. (L.) infantum*, and 20/36 in *L. (V.) braziliensis* infection vs uninfected macrophages (Figure 5). We detected more hypomethylation, mostly associated with miRNA, lncRNA and pseudogenes, than in the CGI analysis. In *L. (L.) infantum* infected samples we detected even 30% differential methylation, such as the promoter at chr6:25246991-25248990 and chr8:12190703-12192702 associated with *KATNBL1P5* and *RNA5SP254* pseudogenes. Due to the definition of promoter [22], one altered genomic region can be associated with different genes. For instance, in *L. (L.) amazonensis* infection, at chromosome 10 two altered promoters, chr10:60027195-60029194 / 60027318-60029317, were associated with *IPMK* and *CISD1* sharing 18 interrogated CpGs, whereas other two regulating regions at chr18:74206647-74208646 / 74205977–74207976 were associated with *ZNF516* and its lncRNA containing 11 and 9 interrogated CpGs. The most altered promoters based on |Δβ| > 0.2 for each species are shown in Tables S5–S7.

## Discussion

We conducted a study focused on a poorly explored area in leishmaniasis that compare the DNA methylation profile among *Leishmania* species including different culture conditions. We first investigated the methylation pattern between uninfected macrophages and macrophages infected with heat-killed *L. (V.) braziliensis*. Although this control does not include the three species, the low number of altered CGIs between both controls suggests that the presence of possible species-specific antigens could not strongly affect the DNA methylation pattern. Moreover, some of these aberrations are also significantly altered in *Leishmania* species. We hypothesize that this effect may be due to either an increased or absence of alteration because of a cooperative or opposite effect of both factors i.e. an active infection and phagocytosis. However, further analysis with heat-killed *L. (L.) amazonensis* and *L. (L.) infantum* are suggested to exclude a probable effect of species-specific thermo-sensitive biomolecules. The following differential methylation analyses between culture conditions and the control were performed using the uninfected macrophages excluding the identified 11 CGIs probably due to phagocytosis.

The similar DNA methylation pattern obtained upon infection with *L. (L.) amazonensis* and *L. (L.) infantum* different from *L. (V.) braziliensis* probably reflects evolutionary history of *Leishmania* since *L. (L.) amazonensis* and *L. (L.) infantum* belong to *Leishmania* subgenus while *L. (V.) braziliensis* to *Viannia* [25]. This contrasts with a previous analysis based on the RNA expression profile of infected macrophages that found *L. (L.) amazonensis* and *L. (V.) braziliensis* more similar than *L. (L.) infantum* [26], suggesting that gene expression depends on other factors besides DNA methylation and may align with the clinical forms. Although our results do not have a direct clinical significance, the identification of species specific and shared DNA methylation alterations provides new potential druggable targets using different approaches including therapies modulating their methylation, gene expression or function. To address this goal, further *in vitro* and *in vivo* functional studies will be conducted to elucidate the consequences of the identified DNA methylation changes at different levels. In our study only two altered CGIs, associated with *ELOVL6* and *EPB41L4B*, were identified in the three species. To the best of our knowledge, only one work reported altered expression of *ELOVL6* upon *L. (L.) major* infection [27].

The three species may overlap in some regions such as in north Argentina [2], thus the identified methylation pattern suggests species-specific interactive strategies with host cells and may contribute to their discrimination in terms of diagnosis, prognosis and treatment. *TRAPPC9*, a vesicular transport component, associated with the most hypomethylated CGI in *L. (L.) amazonensis* infection, was reported dysregulated in *L. (L.) donovani*-infected macrophages [28]. The most hypermethylated CGI in *L. (L.) infantum* infection was associated with a member of the *HLA* Class II genes, the *HLA-DRB5*. Genetic variations at this cluster have been linked to leishmaniasis susceptibility [29]. The identified alteration may contribute to its complex mechanism of gene expression regulation and thus to host response against leishmaniasis. Two members of *TBL1* family were altered in macrophages infected with *L. (L.) infantum*, *TBL1Y* as the most hypomethylated event, and *TBL1XR1*, hypermethylated. Their function is mainly focused on transcriptional regulation [30], but its possible role in *Leishmania* infection is not reported. In *L. (V.) braziliensis* infection the hypomethylated CGI located at *ABCA2*, a member of the superfamily of ATP-binding cassette transporters, presents several variants, suggesting that the methylation alteration may have a role in their regulation. Dysregulation of ABC transporters expression has been reported in macrophages upon *Leishmania* infection, affecting macrophage lipid metabolism, drug internalization/resistance and parasite survival [31]. In our study this methylation signature is further altered upon treatment suggesting *ABCA2* involvement in drug metabolism. However, therapy susceptibility depends on several factors such as host immunity and parasite genetics [3]. In *L. (V.) braziliensis* infected samples, Glucantime treatment further altered the host cell CGI methylation. Several works reported Glucantime-mediated oxidative stress generating reactive oxygen and nitrogen species that interact with DNA, lipids and proteins, and affect superoxide dismutase (SOD) and catalase (CAT) activities [5]. This oxidative environment probably contributes to the altered DNA methylation pattern. In contrast, in *L. (L.) infantum* Amphotericin B-treated samples we observed a DNA methylation restore for some CGIs. It is probable that the treatment period of 48hs was not enough to observe wider methylation aberrations and differences between treatments in terms of drug transport, metabolism and toxicity. Overall, these data show the dynamic behaviour of the DNA methylation pattern in leishmaniasis, its possible re-establishment suggesting DNA methylation alterations as potential therapeutic targets. In the context of infectious diseases epigenetic manipulation is a pioneering research area. For instance, the use of epigenetic therapies in viral infection is currently under investigation from the first experimental trials on human immunodeficiency virus (HIV) epigenetic reprogramming [32]. More recently, Reyser and collaborators underline that the targeting of epigenetic pathways is a promising approach for the treatment of malaria [33].

Gene enrichment analysis demonstrated aberrations in cytoskeleton rearrangement in agreement with previous proteomic studies [34]. Although these protein analyses and our studies were performed at different infection times, GTPases family members such as *CDC42*, *RAC1* and *RHO* were found dysregulated suggesting them as essential players in the infection. Previous evidence reported that this gene family contributes to parasite survival and cell migration in leishmaniasis [35]. Signaling by VEGF that mediates lymphangiogenesis is another identified altered pathway, as previously reported [36]. Regarding the *Leishmania* infection pathway, the altered genes mainly belonging to cytoskeleton dynamics have been already reported in leishmaniasis [37]. To note, within the identified pathways DNA methylation changes occur in some genes suggesting that few genes are targeted for de/methylation probably contributing to each signalling dysregulation.

Since cell and immunological environment may influence the methylome pattern, we included a pretreatment with IL-6 in our experimental assays. In agreement with Almeida *et al*. [9], we did not detect significant differential methylation at *FLI1* between infected samples in presence and absence of IL-6. In the present work the identified alterations were not associated with genes involved in immunity. These results suggest that IL-6-mediated immune response may be regulated at a different step from DNA methylation or longer incubation time might be necessary to observe methylome aberrations. Further studies of IL-6 targets are needed to elucidate its role in our experimental assays.

Our methylome study also showed altered promoters mainly associated with non-coding RNAs in agreement with previously reported dysregulation at the expression level of this RNA category [26]. These results suggest a more complex regulation of macrophage function during *Leishmania*-host interaction.

The present study has strengths and limitations. The infection assays were conducted using a commercial monocyte cell line that may present a different DNA methylation pattern compared to infected cells from patients due to the immortalization process and the subsequent passages in culture [38,39]. This *in vitro* model does not consider environmental factors, essential for cellular crosstalk, such as other cell types and cytokines/chemokines that should be included in a more complex system. Nevertheless, this model confers a relevant contribution of a specific infection period with three species in different culture conditions considering the limited knowledge presently available. Given the importance of CpG-enriched regions during the gene regulation mechanism, our methylation analyses were focused on CGIs and promoters rather than CpG sites as previously performed [8]. Our future studies will test whether selected alterations lead to effective gene expression changes. In addition to the more complex *in vitro* model, described above, we intend to further explore the involvement of the DNA methylation changes in leishmaniasis using cutaneous and visceral *in vivo* models, also considering drug effects on the identified alterations. The animal model will allow to evaluate the association with other variables such as burden parasite and cytokines secretion. Finally, it would be also pivotal to conduct a DNA methylation analysis from samples isolated from patients in order to validate our results.

## Supplementary Material

Supplemental_material_revised-clean.docx
