# Supplementary material for: DNA Methylation in macrophages infected with Leishmania spp. in different culture conditions
Source: Emerg Microbes Infect. 2025 May 21;14(1):2508766. doi: 10.1080/22221751.2025.2508766 (PMC12153010; doi:10.1080/22221751.2025.2508766)
Supplement: Supplemental_material_revised-clean.docx [file TEMI_A_2508766_SM3392.docx]

DNA methylation in macrophages infected with *Leishmania* spp. in different culture conditions

Eleonora Loi^a^; Paola Andrea Barroso^b*^; Agustín Moya Alvarez^b^; Patrizia Zavattari^a^; Ana Florencia Vega Benedetti^a*^

^a^ Department of Biomedical Sciences, Unit of Biology and Genetics, University of Cagliari, 09042 Cagliari, Italy; ^b^ Institute of Experimental Pathology Dr. Miguel Ángel Basombrío - CONICET, University of Salta, 4400 Salta, Argentina

*Correspondence:

Ana Florencia Vega Benedetti, Department of Biomedical Sciences, Biochemistry, Biology and Genetics Unit, University of Cagliari, Cittadella Universitaria di Monserrato SP 8, Km 0.700 - 09042, Monserrato, Cagliari, Italy; Tel. +390706754103; email: anaf.vegab@unica.it;

Paola Andrea Barroso, Institute of Experimental Pathology Dr. Miguel Ángel Basombrío - CONICET, University of Salta, 4400 Salta, Argentina; Tel. +543874255333; email: [barrosopaola75@gmail.com](mailto:barrosopaola75@gmail.com)

**Supplemental material**

**Table S1. Differently methylated CGIs between CtA and CtB and their corresponding Δβ value between CtA and each species.**

|  |  | **CtB/CtA** | | ***L. infantum*/CtA** | | ***L. braziliensis*/CtA** | | ***L. amazonensis*/CtA** | |
| --- | --- | --- | --- | --- | --- | --- | --- | --- | --- |
| **Location** | **Nearest TSS Gene** | **Δβ** | **Nominal p value** | **Δβ** | **Nominal p value** | **Δβ** | **Nominal p value** | **Δβ** | **Nominal p value** |
| **chr1:32892429-32892835** | ***BSDC1*** | **-0.123** | **0.002** | **-0.140** | **0.004** | **-0.071** | **0.391** | **-0.183** | **0.106** |
| **chr10:132583721-132584164** | ***MIR378C*** | **0.117** | **0.011** | **0.030** | **0.299** | **0.054** | **0.071** | **0.076** | **0.063** |
| **chr11:858576-858932** | ***TSPAN4*** | **0.225** | **0.002** | **0.091** | **0.022** | **0.245** | **4.81E-05** | **0.078** | **0.473** |
| **chr12:6165942-6166257** | ***VWF*** | **-0.113** | **0.002** | **-0.235** | **3.42E-05** | **-0.151** | **3.01E-04** | **-0.277** | **1.27E-04** |
| **chr14:105499803-105500021** | ***CDCA4*** | **0.144** | **0.005** | **0.091** | **0.033** | **0.110** | **0.007** | **0.152** | **0.002** |
| **chr16:87894360-87894566** | ***SLC7A5*** | **-0.308** | **0.028** | **-0.138** | **0.021** | **-0.200** | **0.030** | **-0.008** | **0.075** |
| **chr16:88442450-88442994** | ***ZNF469*** | **-0.175** | **2.85E-04** | **0.042** | **0.028** | **-0.029** | **0.059** | **0.015** | **0.021** |
| **chr22:43165948-43166287** | ***A4GALT*** | **-0.473** | **1.31E-06** | **-0.005** | **0.148** | **0.006** | **0.114** | **0.005** | **0.164** |
| **chr5:179517443-179517664** | ***RNF130*** | **0.112** | **0.015** | **0.183** | **0.002** | **0.182** | **0.001** | **0.144** | **0.017** |
| **chr9:77111779-77112042** | ***RORB*** | **-0.224** | **0.010** | **-0.088** | **0.269** | **-0.198** | **0.023** | **-0.156** | **0.037** |
| **chrY:28555536-28555932** | ***TTTY3*** | **-0.124** | **0.049** | **-0.042** | **0.163** | **0.000** | **0.875** | **0.010** | **0.635** |

Note: uninfected macrophages (CtA: control A), macrophages with heat-killed *Leishmania* (CtB: control B) transcription start site (TSS).

**Table S2**. *L. (L.) amazonensis*-specific altered CGIs.

| **Location** | **Δβ *L. amazonensis*/CtA** | **Nominal p value** | **Nearest TSS Gene** |
| --- | --- | --- | --- |
| chr7:149469543-149469748 | 0.174 | 0.006 | *ZNF467* |
| chr9:98273679-98273908 | 0.154 | 0.004 | *PTCH1* |
| chr19:2349979-2350487 | 0.151 | 0.004 | *SPPL2B* |
| chr11:518683-520297 | 0.150 | 0.002 | *RNH1* |
| chr2:71192095-71192495 | 0.149 | 0.009 | *ANKRD53* |
| chr18:75886578-75886801 | 0.147 | 0.003 | *SALL3* |
| chr9:136426125-136426390 | 0.144 | 0.001 | *ADAMTSL2* |
| chr9:14978095-14978585 | 0.144 | 0.022 | *LOC389705* |
| chr7:106809462-106809815 | 0.143 | 0.005 | *HBP1* |
| chr2:174877566-174877778 | 0.142 | 0.008 | *SP3* |
| chr19:11491560-11492740 | 0.142 | 0.007 | *EPOR* |
| chr10:134774214-134774485 | 0.142 | 0.002 | *LINC01168* |
| chr17:78954994-78955381 | 0.140 | 0.003 | *CHMP6* |
| chr19:53360825-53361067 | 0.140 | 0.006 | *ZNF468* |
| chr9:77113710-77113927 | 0.139 | 0.010 | *RORB* |
| chr6:19837015-19837228 | 0.138 | 0.016 | *ID4* |
| chr2:232063174-232063616 | 0.137 | 0.003 | *ARMC9* |
| chr8:17013437-17014790 | 0.136 | 0.005 | *ZDHHC2* |
| chr20:1276888-1277121 | 0.135 | 0.014 | *SDCBP2* |
| chr20:32254812-32255989 | 0.135 | 0.010 | *ACTL10* |
| chr20:60494769-60495449 | 0.134 | 0.007 | *MIR1257* |
| chr2:92230349-92231783 | 0.133 | 0.006 | *ACTR3BP2* |
| chr12:39300470-39300680 | 0.133 | 0.006 | *CPNE8* |
| chr7:63222280-63222688 | 0.133 | 0.021 | *MIR4283-1* |
| chr19:8661081-8661287 | 0.132 | 0.001 | *ADAMTS10* |
| chr18:43355319-43355698 | 0.132 | 0.006 | *SLC14A1* |
| chr2:163200576-163201025 | 0.132 | 0.004 | *GCA* |
| chr13:113745162-113745644 | 0.131 | 0.017 | *F7* |
| chr14:24808636-24808992 | 0.131 | 0.016 | *RIPK3* |
| chr17:40827976-40828659 | 0.130 | 0.010 | *PLEKHH3* |
| chr6:27440614-27441065 | 0.129 | 0.011 | *ZNF184* |
| chr2:172373818-172374199 | 0.129 | 0.012 | *CYBRD1* |
| chr7:151722591-151723241 | 0.129 | 0.006 | *GALNT11* |
| chr4:74702421-74702627 | 0.129 | 0.009 | *CXCL6* |
| chr19:12876890-12877108 | 0.129 | 0.006 | *HOOK2* |
| chr5:156277303-156277685 | 0.129 | 0.011 | *PPP1R2B* |
| chr13:53424942-53425995 | 0.129 | 0.014 | *PCDH8* |
| chr20:50178961-50179675 | 0.129 | 0.006 | *NFATC2* |
| chr7:948295-949168 | 0.129 | 0.005 | *ADAP1* |
| chr7:64601086-64601891 | 0.128 | 0.032 | *CCT6P3* |
| chr1:16163753-16164123 | 0.128 | 0.009 | *SPEN* |
| chr15:40697782-40698368 | 0.128 | 0.007 | *IVD* |
| chr8:77590168-77590373 | 0.128 | 0.010 | *ZFHX4* |
| chr22:50328694-50329178 | 0.127 | 0.020 | *CRELD2* |
| chrX:25038891-25039204 | 0.127 | 0.009 | *ARX* |
| chr6:39869580-39869800 | 0.127 | 0.001 | *MOCS1* |
| chr18:75577-76027 | 0.127 | 0.013 | *ROCK1P1* |
| chr22:31318229-31318489 | 0.126 | 0.009 | *MORC2-AS1* |
| chr4:55094322-55094593 | 0.125 | 0.009 | *PDGFRA* |
| chr19:50931271-50931638 | 0.124 | 0.003 | *MYBPC2* |
| chr5:122971759-122972008 | 0.123 | 0.008 | *CSNK1G3* |
| chr22:42078061-42078549 | 0.123 | 0.012 | *SNU13* |
| chr6:73972820-73973027 | 0.122 | 0.018 | *KHDC1* |
| chr9:68809169-68810258 | 0.122 | 0.010 | *FRG1HP* |
| chr19:4759819-4760070 | 0.122 | 0.013 | *MIR7-3HG* |
| chr18:56975843-56976160 | 0.122 | 0.016 | *CPLX4* |
| chr6:158957221-158958677 | 0.121 | 0.003 | *TMEM181* |
| chr20:50108796-50109456 | 0.121 | 0.005 | *MIR3194* |
| chr19:23386650-23387069 | 0.120 | 0.003 | *ZNF724* |
| chr19:19335772-19336151 | 0.120 | 0.009 | *NCAN* |
| chr1:3182822-3183216 | 0.120 | 0.029 | *MIR4251* |
| chr1:38259096-38260427 | 0.120 | 0.009 | *MANEAL* |
| chr11:55641711-55642120 | 0.119 | 0.040 | *TRIM51* |
| chr10:28287448-28288057 | 0.119 | 0.004 | *ODAD2* |
| chr16:71392416-71392749 | 0.119 | 0.011 | *CALB2* |
| chrX:111623832-111624042 | 0.119 | 0.043 | *LHFPL1* |
| chr4:39172243-39172522 | 0.119 | 0.021 | *WDR19* |
| chr8:62051647-62052431 | 0.118 | 0.007 | *CLVS1* |
| chr7:89874322-89874855 | 0.118 | 0.006 | *CFAP69* |
| chr7:28448717-28450028 | 0.118 | 0.010 | *CREB5* |
| chr2:159824855-159826401 | 0.118 | 0.001 | *TANC1* |
| chr6:46620542-46621189 | 0.118 | 0.007 | *SLC25A27* |
| chr15:72929134-72929578 | 0.118 | 0.045 | *GOLGA6B* |
| chr1:228295369-228295569 | 0.118 | 0.001 | *MRPL55* |
| chr4:17513310-17513981 | 0.118 | 0.007 | *QDPR* |
| chr9:130007230-130007688 | 0.117 | 0.010 | *GARNL3* |
| chr1:237947188-237947448 | 0.117 | 0.014 | *RYR2* |
| chr19:21265165-21265433 | 0.117 | 0.020 | *ZNF714* |
| chr9:137719284-137719504 | 0.117 | 0.016 | *MIR3689C* |
| chr15:22692687-22693187 | 0.117 | 0.021 | *MIR4509-1* |
| chr16:32822902-32823585 | 0.117 | 0.009 | *SLC6A10P* |
| chr7:29233705-29235046 | 0.116 | 0.005 | *CPVL* |
| chr10:28965705-28967266 | 0.116 | 0.006 | *BAMBI* |
| chr5:66299769-66300083 | 0.116 | 0.014 | *MAST4* |
| chr20:61456340-61456565 | 0.116 | 0.012 | *COL9A3* |
| chr1:86042230-86042935 | 0.116 | 0.009 | *DDAH1* |
| chr3:107149821-107150910 | 0.116 | 0.011 | *CCDC54* |
| chr18:22006311-22007007 | 0.116 | 0.008 | *IMPACT* |
| chr10:134607424-134607880 | 0.116 | 0.025 | *NKX6-2* |
| chr13:102106166-102106488 | 0.115 | 0.006 | *ITGBL1* |
| chr10:115933380-115934262 | 0.115 | 0.009 | *MIR2110* |
| chr15:51973534-51973838 | 0.115 | 0.003 | *SCG3* |
| chr1:142890555-142891041 | 0.115 | 0.024 | *ANKRD20A12P* |
| chr12:12849068-12849270 | 0.115 | 0.004 | *GPR19* |
| chr1:15479220-15481471 | 0.114 | 0.004 | *TMEM51* |
| chr20:633542-634330 | 0.114 | 0.009 | *SRXN1* |
| chr1:144519970-144521503 | 0.114 | 0.010 | *NA* |
| chr9:33510859-33511221 | 0.114 | 0.017 | *SUGT1P1* |
| chr7:27203916-27206462 | 0.113 | 0.006 | *HOXA9* |
| chr1:159158229-159158498 | 0.113 | 0.007 | *CADM3-AS1* |
| chr17:18528377-18528714 | 0.113 | 0.008 | *CCDC144BP* |
| chr1:33938026-33938328 | 0.113 | 0.009 | *ZSCAN20* |
| chr13:78271802-78272906 | 0.113 | 0.008 | *SLAIN1* |
| chr19:30334441-30334688 | 0.113 | 0.028 | *CCNE1* |
| chrX:47478671-47479515 | 0.113 | 0.011 | *SYN1* |
| chr16:1309158-1309415 | 0.113 | 0.006 | *TPSD1* |
| chr5:139493596-139494541 | 0.113 | 0.002 | *PURA* |
| chr15:28982670-28983497 | 0.113 | 0.007 | *WHAMMP2* |
| chr9:129276572-129276977 | 0.113 | 0.012 | *LMX1B* |
| chr9:41954479-41954842 | 0.112 | 0.025 | *GLIDR* |
| chr4:148652849-148653918 | 0.112 | 0.008 | *ARHGAP10* |
| chr2:105483754-105484426 | 0.112 | 0.004 | *POU3F3* |
| chr5:95296369-95297438 | 0.112 | 0.008 | *ELL2* |
| chr19:17346166-17346886 | 0.112 | 0.015 | *OCEL1* |
| chr18:6729694-6730037 | 0.112 | 0.002 | *ARHGAP28* |
| chr3:98620132-98620910 | 0.112 | 0.009 | *DCBLD2* |
| chr20:1206680-1207119 | 0.112 | 0.005 | *RAD21L1* |
| chr6:158652706-158652946 | 0.111 | 0.016 | *GTF2H5* |
| chr3:46853728-46854138 | 0.111 | 0.007 | *PRSS50* |
| chrX:151649043-151649487 | 0.111 | 0.009 | *GABRA3* |
| chr20:16555519-16555779 | 0.111 | 0.015 | *KIF16B* |
| chr15:41165848-41166571 | 0.111 | 0.005 | *RHOV* |
| chr20:62737383-62738071 | 0.111 | 0.005 | *NPBWR2* |
| chr16:58549028-58550016 | 0.111 | 0.013 | *SETD6* |
| chr9:45356018-45356994 | 0.111 | 0.024 | *FAM27C* |
| chr1:149575949-149577505 | 0.111 | 0.002 | *LINC00869* |
| chr7:32981939-32982865 | 0.111 | 0.023 | *RP9P* |
| chr15:65197800-65198063 | 0.111 | 0.013 | *ANKDD1A* |
| chr9:22446682-22447695 | 0.111 | 0.011 | *DMRTA1* |
| chr21:42539368-42540872 | 0.111 | 0.007 | *BACE2* |
| chr22:16867375-16868185 | 0.111 | 0.022 | *CCT8L2* |
| chr4:58061752-58062115 | 0.111 | 0.047 | *IGFBP7* |
| chr6:27833121-27833406 | 0.111 | 0.019 | *H2AC16* |
| chr7:644182-644461 | 0.111 | 0.010 | *PRKAR1B* |
| chr5:39424893-39425320 | 0.111 | 0.016 | *DAB2* |
| chr12:83080663-83081646 | 0.111 | 0.010 | *TMTC2* |
| chr21:28515803-28516306 | 0.111 | 0.029 | *ADAMTS5* |
| chr5:122371570-122372822 | 0.111 | 0.013 | *PPIC* |
| chr5:179554111-179554556 | 0.111 | 0.002 | *RASGEF1C* |
| chr16:89988831-89989746 | 0.110 | 0.018 | *TUBB3* |
| chr12:100378244-100378582 | 0.110 | 0.009 | *ANKS1B* |
| chr20:49457138-49457342 | 0.110 | 0.027 | *BCAS4* |
| chr15:37393667-37394248 | 0.110 | 0.002 | *MEIS2* |
| chr14:64805110-64805785 | 0.110 | 0.009 | *ESR2* |
| chr1:53308295-53309262 | 0.110 | 0.004 | *ZYG11A* |
| chr9:39817325-39817937 | 0.110 | 0.020 | *NA* |
| chr5:140764302-140764680 | 0.110 | 0.007 | *PCDHGA7* |
| chr1:182758396-182759097 | 0.110 | 0.007 | *NPL* |
| chr12:104609398-104610172 | 0.110 | 0.008 | *TXNRD1* |
| chr13:112715360-112716234 | 0.110 | 0.009 | *SOX1* |
| chr3:32147827-32148874 | 0.109 | 0.012 | *GPD1L* |
| chr2:10572392-10572752 | 0.109 | 0.000 | *SNORA80B* |
| chr9:34377402-34377610 | 0.109 | 0.029 | *MYORG* |
| chr8:21867733-21868204 | 0.109 | 0.004 | *NPM2* |
| chr7:97501184-97501919 | 0.109 | 0.001 | *ASNS* |
| chr12:75784800-75785241 | 0.109 | 0.015 | *GLIPR1L2* |
| chr1:119543822-119544339 | 0.109 | 0.002 | *TBX15* |
| chr2:204399491-204400179 | 0.109 | 0.017 | *RAPH1* |
| chr1:1389556-1389889 | 0.109 | 0.003 | *ATAD3C* |
| chr11:130318235-130320198 | 0.109 | 0.013 | *ADAMTS15* |
| chr5:41869664-41870876 | 0.109 | 0.017 | *OXCT1* |
| chr5:57878726-57879177 | 0.109 | 0.015 | *RAB3C* |
| chr5:140787448-140788044 | 0.109 | 0.011 | *PCDHGB6* |
| chrY:21154604-21155040 | 0.109 | 0.009 | *CD24* |
| chr19:33764350-33764551 | 0.108 | 0.019 | *CEBPA* |
| chr3:155027499-155027703 | 0.108 | 0.002 | *STRIT1* |
| chr2:110370907-110373301 | 0.108 | 0.005 | *SOWAHC* |
| chr2:132431211-132431768 | 0.108 | 0.011 | *C2orf27A* |
| chr5:143584744-143585064 | 0.108 | 0.008 | *KCTD16* |
| chr16:3199653-3199937 | 0.108 | 0.026 | *ZNF213* |
| chr11:119227097-119227758 | 0.108 | 0.015 | *USP2* |
| chr1:205631978-205632254 | 0.108 | 0.002 | *SLC45A3* |
| chr2:100720386-100720741 | 0.108 | 0.044 | *AFF3* |
| chr18:12307555-12309043 | 0.107 | 0.003 | *TUBB6* |
| chrY:13370337-13370718 | 0.107 | 0.030 | *GYG2P1* |
| chr4:41215872-41216813 | 0.107 | 0.011 | *APBB2* |
| chr12:125670118-125672112 | 0.107 | 0.014 | *AACS* |
| chr16:32264532-32265444 | 0.107 | 0.011 | *TP53TG3D* |
| chr3:105072530-105072962 | 0.107 | 0.012 | *ALCAM* |
| chr17:48132995-48134084 | 0.107 | 0.007 | *ITGA3* |
| chr7:5526390-5526693 | 0.107 | 0.036 | *MIR589* |
| chr19:53935555-53935823 | 0.107 | 0.046 | *TPM3P9* |
| chr11:124932731-124933657 | 0.107 | 0.019 | *SLC37A2* |
| chr20:44440947-44441655 | 0.107 | 0.010 | *UBE2C* |
| chr5:137667503-137668037 | 0.107 | 0.021 | *CDC25C* |
| chr10:47151425-47151676 | 0.107 | 0.015 | *LINC00842* |
| chr2:62732759-62733497 | 0.107 | 0.016 | *TMEM17* |
| chr19:58549306-58549712 | 0.107 | 0.019 | *ZSCAN1* |
| chr22:50708191-50710490 | 0.107 | 0.011 | *MAPK11* |
| chr12:132974468-132974675 | 0.106 | 0.016 | *GALNT9* |
| chr14:58618292-58619220 | 0.106 | 0.009 | *ARMH4* |
| chr19:2900330-2901203 | 0.106 | 0.002 | *ZNF57* |
| chr19:53696030-53696650 | 0.106 | 0.024 | *ZNF665* |
| chr17:21318651-21320041 | 0.106 | 0.008 | *KCNJ12* |
| chr19:51505915-51506363 | 0.106 | 0.016 | *KLK8* |
| chr20:60448776-60449063 | 0.106 | 0.010 | *MIR1257* |
| chr4:128702831-128704215 | 0.106 | 0.016 | *HSPA4L* |
| chr1:148241110-148241449 | 0.106 | 0.021 | *NBPF14* |
| chr18:21977276-21978110 | 0.106 | 0.020 | *OSBPL1A* |
| chr12:132848095-132848790 | 0.106 | 0.004 | *LOC100130238* |
| chr14:104569500-104569780 | 0.106 | 0.001 | *MIR203A* |
| chr1:135125-135563 | 0.106 | 0.012 | *LOC729737* |
| chr11:43702017-43702597 | 0.105 | 0.014 | *HSD17B12* |
| chr1:244013803-244014592 | 0.105 | 0.006 | *AKT3* |
| chr9:141044509-141045113 | 0.105 | 0.006 | *TUBBP5* |
| chr15:96900143-96900644 | 0.105 | 0.022 | *NR2F2* |
| chr1:45251963-45252292 | 0.105 | 0.033 | *BEST4* |
| chr16:3232836-3234048 | 0.105 | 0.018 | *OR1F1* |
| chr17:73749619-73750178 | 0.105 | 0.006 | *GALK1* |
| chr6:27835191-27835461 | 0.105 | 0.008 | *H1-5* |
| chr3:98451287-98452225 | 0.105 | 0.022 | *ST3GAL6* |
| chr5:125759048-125759298 | 0.105 | 0.029 | *GRAMD2B* |
| chr2:132795200-132796196 | 0.105 | 0.019 | *ANKRD30BL* |
| chr6:75911580-75912133 | 0.105 | 0.013 | *COL12A1* |
| chr19:4607080-4607350 | 0.105 | 0.000 | *TNFAIP8L1* |
| chr13:112212063-112212297 | 0.104 | 0.015 | *TEX29* |
| chr21:39287947-39289267 | 0.104 | 0.010 | *KCNJ6* |
| chr7:23508185-23509712 | 0.104 | 0.019 | *IGF2BP3* |
| chr14:74079184-74079657 | 0.104 | 0.017 | *ACOT6* |
| chr2:71680559-71681276 | 0.104 | 0.007 | *DYSF* |
| chr20:58179809-58180787 | 0.104 | 0.014 | *PHACTR3* |
| chr1:161953617-161954039 | 0.104 | 0.007 | *OLFML2B* |
| chr10:60027008-60029241 | 0.104 | 0.017 | *CISD1* |
| chr6:134159127-134159349 | 0.104 | 0.040 | *LINC01312* |
| chr16:48844552-48845264 | 0.104 | 0.012 | *N4BP1* |
| chr9:98514400-98514729 | 0.104 | 0.021 | *ERCC6L2* |
| chr3:126108020-126108488 | 0.104 | 0.040 | *CFAP100* |
| chr12:131417943-131418218 | 0.104 | 0.016 | *ADGRD1* |
| chr16:68563760-68564281 | 0.104 | 0.023 | *ZFP90* |
| chr19:1854469-1854863 | 0.104 | 0.015 | *REXO1* |
| chr19:57078366-57079301 | 0.104 | 0.013 | *ZNF470* |
| chr8:81083321-81084057 | 0.104 | 0.003 | *TPD52* |
| chr10:134682701-134682917 | 0.104 | 0.034 | *CFAP46* |
| chr2:171627226-171628062 | 0.103 | 0.004 | *NA* |
| chr3:142442703-142443978 | 0.103 | 0.013 | *TRPC1* |
| chr6:146055897-146056981 | 0.103 | 0.020 | *EPM2A-DT* |
| chr2:88316234-88316859 | 0.103 | 0.022 | *RGPD1* |
| chr15:85113436-85114361 | 0.103 | 0.006 | *UBE2Q2P1* |
| chr21:46448083-46448593 | 0.103 | 0.050 | *PICSAR* |
| chr11:36397927-36399398 | 0.103 | 0.029 | *PRR5L* |
| chr7:23513535-23514412 | 0.103 | 0.017 | *IGF2BP3* |
| chr10:75118206-75118679 | 0.103 | 0.008 | *CFAP70* |
| chr6:56818874-56820308 | 0.103 | 0.018 | *DST* |
| chr5:174162367-174162843 | 0.103 | 0.003 | *MSX2* |
| chr19:58595817-58596927 | 0.103 | 0.013 | *ZSCAN18* |
| chr9:138193485-138193805 | 0.103 | 0.023 | *LINC02907* |
| chr7:54731780-54732479 | 0.103 | 0.050 | *SEC61G* |
| chr2:239140097-239140659 | 0.103 | 0.013 | *TARDBPP3* |
| chr3:138153270-138154621 | 0.103 | 0.011 | *ESYT3* |
| chr6:72294373-72294988 | 0.103 | 0.010 | *LINC00472* |
| chr1:112938496-112939113 | 0.103 | 0.005 | *CTTNBP2NL* |
| chr19:37957727-37958390 | 0.102 | 0.017 | *ZNF569* |
| chr19:23456575-23456868 | 0.102 | 0.017 | *ZNF724* |
| chr3:53194717-53196133 | 0.102 | 0.020 | *PRKCD* |
| chr1:117487250-117487645 | 0.102 | 0.000 | *PTGFRN* |
| chr6:30523287-30523519 | 0.102 | 0.007 | *GNL1* |
| chr17:48624416-48624723 | 0.102 | 0.015 | *SPATA20* |
| chr14:68086278-68086957 | 0.102 | 0.011 | *ARG2* |
| chr17:46654054-46654369 | 0.102 | 0.009 | *HOXB4* |
| chr19:51227662-51228883 | 0.102 | 0.013 | *CLEC11A* |
| chr20:6748053-6749546 | 0.102 | 0.011 | *BMP2* |
| chr16:67564059-67564332 | 0.102 | 0.002 | *RIPOR1* |
| chr9:38067684-38069628 | 0.102 | 0.003 | *SHB* |
| chr2:96314996-96315230 | 0.102 | 0.033 | *TRIM43* |
| chr1:39570666-39571859 | 0.102 | 0.022 | *MACF1* |
| chr10:22764709-22767050 | 0.102 | 0.023 | *LINC03027* |
| chr11:1958935-1959247 | 0.102 | 0.033 | *MRPL23* |
| chr10:12390803-12392589 | 0.102 | 0.036 | *CAMK1D* |
| chr10:101293016-101293238 | 0.102 | 0.019 | *NKX2-3* |
| chrY:16941823-16942188 | 0.102 | 0.003 | *NLGN4Y-AS1* |
| chr6:35772961-35773172 | 0.102 | 0.010 | *LHFPL5* |
| chr11:71524540-71524853 | 0.101 | 0.015 | *ALG1L9P* |
| chr7:5862831-5863066 | 0.101 | 0.023 | *ZNF815P* |
| chr14:107221093-107221298 | 0.101 | 0.009 | *LINC00221* |
| chr4:174459201-174460054 | 0.101 | 0.018 | *HAND2-AS1* |
| chr9:137730887-137731202 | 0.101 | 0.013 | *MIR3689C* |
| chr6:97372182-97372661 | 0.101 | 0.017 | *KLHL32* |
| chr1:144339721-144341289 | 0.101 | 0.019 | *LINC00623* |
| chr13:19183084-19183959 | 0.101 | 0.008 | *LINC00417* |
| chr16:56701864-56702208 | 0.101 | 0.022 | *MT1G* |
| chr22:30278979-30279858 | 0.101 | 0.016 | *MTMR3* |
| chr11:8284103-8285032 | 0.101 | 0.012 | *LMO1* |
| chr1:40156931-40158514 | 0.101 | 0.005 | *HPCAL4* |
| chr8:49890387-49890874 | 0.101 | 0.013 | *SNAI2* |
| chr12:108522951-108523852 | 0.101 | 0.009 | *WSCD2* |
| chr2:203879250-203879891 | 0.101 | 0.018 | *NBEAL1* |
| chr10:133930596-133931010 | 0.101 | 0.009 | *JAKMIP3* |
| chr16:89267824-89268087 | 0.101 | 0.020 | *SLC22A31* |
| chr15:73343780-73345369 | 0.101 | 0.010 | *NEO1* |
| chr21:40032244-40033665 | 0.101 | 0.007 | *ERG* |
| chr1:44871110-44874047 | 0.101 | 0.024 | *RNF220* |
| chr10:44143857-44144698 | 0.101 | 0.019 | *ZNF32* |
| chr1:209920954-209921345 | 0.101 | 0.023 | *TRAF3IP3* |
| chr14:74684442-74684676 | 0.101 | 0.040 | *VSX2* |
| chr15:34806491-34807382 | 0.101 | 0.013 | *MIR1233-2* |
| chr18:74203539-74205017 | 0.101 | 0.009 | *ZNF516* |
| chr19:12098572-12098950 | 0.101 | 0.027 | *ZNF763* |
| chr20:62318360-62318658 | 0.100 | 0.018 | *RTEL1-TNFRSF6B* |
| chr19:51142285-51142986 | 0.100 | 0.016 | *SYT3* |
| chr19:468707-468950 | 0.100 | 0.018 | *ODF3L2* |
| chr8:140631097-140631332 | 0.100 | 0.010 | *KCNK9* |
| chr6:46702737-46703316 | 0.100 | 0.001 | *PLA2G7* |
| chr1:200707850-200708948 | 0.100 | 0.010 | *CAMSAP2* |
| chr12:58004983-58005351 | 0.100 | 0.003 | *ARHGEF25* |
| chr6:128901554-128901925 | 0.100 | 0.002 | *PTPRK* |
| chr20:6103437-6103970 | 0.100 | 0.026 | *FERMT1* |
| chr10:22540708-22542739 | 0.100 | 0.032 | *LOC100130992* |
| chr5:179004498-179004836 | -0.101 | 0.000 | *RUFY1* |
| chr6:169309818-169310582 | -0.101 | 0.017 | *SMOC2* |
| chr20:60911976-60912249 | -0.102 | 0.010 | *MIR4758* |
| chr19:39226726-39226983 | -0.102 | 0.000 | *CAPN12* |
| chr1:154989750-154990234 | -0.103 | 0.036 | *ZBTB7B* |
| chr19:56041124-56041791 | -0.105 | 0.030 | *SBK2* |
| chr14:106138655-106139261 | -0.105 | 0.001 | *ELK2AP* |
| chr7:63216955-63217363 | -0.117 | 0.006 | *MIR4283-1* |
| chrY:20508191-20508452 | -0.124 | 0.014 | *FAM224B* |
| chr3:194785978-194786549 | -0.129 | 0.011 | *XXYLT1* |
| chr11:943738-944142 | -0.150 | 0.026 | *AP2A2* |
| chrY:19671325-19671587 | -0.172 | 0.044 | *FAM224B* |
| chr8:141359156-141359621 | -0.174 | 0.003 | *TRAPPC9* |

Note: uninfected macrophages (CtA: control A), transcription start site (TSS).

**Table S3**. *L. (L.) infantum*-specific altered CGIs.

| **Location** | **Δβ *L. infantum*/CtA** | **Nominal p value** | **Nearest TSS Gene** |
| --- | --- | --- | --- |
| chr6:32489743-32490128 | 0.147693682 | 0.000101168 | *HLA-DRB5* |
| chr19:22444442-22444715 | 0.135875954 | 0.042075791 | *ZNF729* |
| chr16:15221569-15222202 | 0.114778776 | 0.001256877 | *MIR3180-4* |
| chr19:1861712-1861914 | 0.114082688 | 0.014647751 | *KLF16* |
| chr2:211035479-211036637 | 0.114048111 | 0.009850059 | *KANSL1L* |
| chr9:140111750-140111992 | 0.112224152 | 7.00E-06 | *RNF208* |
| chr11:64863699-64863980 | 0.111677684 | 0.003511197 | *VPS51* |
| chr2:160654230-160654631 | 0.110891129 | 0.004668132 | *CD302* |
| chr5:52775534-52776645 | 0.110017087 | 0.009651851 | *FST* |
| chr3:127347683-127348859 | 0.109382005 | 0.000747159 | *PODXL2* |
| chr22:46777707-46777929 | 0.107930051 | 0.025082068 | *CELSR1* |
| chr15:101938646-101938873 | 0.107619318 | 0.000102939 | *PCSK6* |
| chr2:33952423-33952684 | 0.10555264 | 0.00680764 | *MYADML* |
| chr5:79286585-79287134 | 0.104854064 | 0.007081345 | *MTX3* |
| chr22:22311565-22311819 | 0.104825668 | 0.001162597 | *PPM1F* |
| chr11:63993590-63993834 | 0.103911353 | 0.013577355 | *NUDT22* |
| chr17:15466360-15466843 | 0.103737995 | 0.005641818 | *TVP23C-CDRT4* |
| chr6:3224917-3225634 | 0.103636985 | 2.70E-05 | *TUBB2B* |
| chr1:3511710-3512118 | 0.103536308 | 0.000603807 | *MEGF6* |
| chr8:143609379-143609602 | 0.103252065 | 0.004606431 | *ADGRB1* |
| chr22:42353660-42353900 | 0.10125995 | 0.001511435 | *SMIM45* |
| chr16:66304286-66304631 | 0.100960559 | 0.007560098 | *CDH5* |
| chr3:8543202-8543579 | 0.100131638 | 0.003978288 | *LMCD1* |
| chr1:65613042-65614898 | 0.100003818 | 0.001815766 | *AK4* |
| chr16:89173705-89173905 | -0.101522639 | 0.000113111 | *ACSF3* |
| chr16:84346477-84346931 | -0.104444531 | 0.001802726 | *WFDC1* |
| chr11:993209-994006 | -0.104619141 | 0.001169538 | *AP2A2* |
| chr9:116918300-116918522 | -0.122879917 | 0.047475838 | *COL27A1* |
| chr22:45182017-45182223 | -0.125807076 | 0.003035862 | *ARHGAP8* |
| chr2:217674966-217675214 | -0.130598217 | 0.009591452 | *TNP1* |
| chr16:3085223-3085469 | -0.136890935 | 0.000392429 | *BICDL2* |
| chr3:13245693-13246212 | -0.147457479 | 5.52E-05 | *IQSEC1* |
| chr20:60553116-60553599 | -0.15710961 | 0.0004587 | *MIR1257* |
| chrY:19691215-19691762 | -0.157505519 | 0.003970887 | *FAM224B* |
| chrY:6778575-6780028 | -0.182207423 | 0.000581793 | *TBL1Y* |

Note: uninfected macrophages (CtA: control A), transcription start site (TSS).

**Table S4**. *L. (V.) braziliensis*-specific altered CGIs.

| **Location** | **Δβ *L. braziliensis*/CtA** | **Nominal p value** | **Nearest TSS Gene** |
| --- | --- | --- | --- |
| chr8:1350140-1350577 | 0.103 | 0.005 | *DLGAP2* |
| chr9:138011607-138011815 | 0.111 | 0.038 | *OLFM1* |
| chr9:139917179-139917492 | -0.174 | 1.67E-05 | *ABCA2* |

Note: uninfected macrophages (CtA: control A), transcription start site (TSS).

**Table S5**. *L. (L.) amazonensis*-specific altered promoters.

| **Location** | **Δβ *L. amazonensis*/CtA** | **Nominal p value** | **Nearest TSS gene** | **Ensembl ID** |
| --- | --- | --- | --- | --- |
| chr1:219615485-219617484 | -0.252 | 4.77E-05 | NA | ENSG00000230024 |
| chr12:101802339-101804338 | -0.233 | 0.035 | NA | ENSG00000257543 |
| chr15:96607156-96609155 | -0.215 | 0.001 | NA | ENSG00000259702 |
| chr15:44682150-44684149 | -0.262 | 0.004 | NA | ENSG00000259499 |
| chr17:62567447-62569446 | -0.213 | 1.58E-05 | NA | ENSG00000271779 |
| chr2:228677058-228679057 | -0.205 | 3.04E-04 | *CCL20* | ENSG00000115009 |
| chr2:231564745-231566744 | -0.229 | 0.023 | NA | ENSG00000226125 |
| chr22:41562721-41564720 | -0.217 | 0.016 | *RNU6-375P* | ENSG00000252859 |
| chr22:17306864-17308863 | -0.218 | 0.022 | *HSFY1P1* | ENSG00000229027 |
| chr3:177725068-177727067 | -0.237 | 0.022 | *RNU6-1120P* | ENSG00000199858 |
| chr5:1308993-1310992 | -0.207 | 0.012 | *MIR4457* | ENSG00000263670 |
| chr5:180681220-180683219 | -0.223 | 0.003 | NA | ENSG00000268397 |
| chr6:25246991-25248990 | -0.207 | 0.002 | *KATNBL1P5* | ENSG00000271108 |
| chr7:140086208-140088207 | -0.204 | 0.006 | *RNA5SP248* | ENSG00000202472 |
| chr8:48438459-48440458 | 0.225 | 6.65E-05 | NA | ENSG00000269924 |

Note: uninfected macrophages (CtA: control A), transcription start site (TSS), no available data (NA).

**Table S6**. *L. (L.) infantum*-specific altered promoters.

| **Location** | **Δβ *L. infantum*/CtA** | **Nominal p value** | **Nearest TSS gene** | **Ensembl ID** |
| --- | --- | --- | --- | --- |
| chr3:49843179-49845178 | 0.237 | 4.17E-04 | *MIR5193* | ENSG00000263506 |
| chr9:27005192-27007191 | -0.205 | 0.001 | *LRRC19* | ENSG00000184434 |
| chr11:59843323-59845322 | -0.208 | 0.001 | NA | ENSG00000255331 |
| chr15:44682150-44684149 | -0.212 | 0.002 | NA | ENSG00000259499 |
| chr3:180297345-180299344 | -0.222 | 0.001 | RNU6-486P | ENSG00000199986 |
| chr3:39398536-39400535 | -0.226 | 0.001 | *EEF1A1P24* | ENSG00000223668 |
| chr1:219615485-219617484 | -0.230 | 1.77E-05 | NA | ENSG00000230024 |
| chr1:59754813-59756812 | -0.232 | 6.21E-07 | NA | ENSG00000270457 |
| chr3:177725068-177727067 | -0.256 | 0.006 | RNU6-1120P | ENSG00000199858 |
| chr12:112739968-112741967 | -0.258 | 5.05E-05 | RPL7AP60 | ENSG00000213152 |
| chr9:5106437-5108436 | -0.260 | 5.22E-05 | *MTND4P14* | ENSG00000236254 |
| chrX:21889325-21891324 | -0.289 | 0.001 | NA | ENSG00000206639 |
| chr6:25246991-25248990 | -0.300 | 1.39E-04 | *KATNBL1P5* | ENSG00000271108 |
| chr8:12190703-12192702 | -0.302 | 6.80E-05 | *RNA5SP254* | ENSG00000252535 |

Note: uninfected macrophages (CtA: control A), transcription start site (TSS), no available data (NA).

**Table S7**. *L. (V.) braziliensis*-specific altered promoters.

| **Location** | **Δβ *L. braziliensis*/CtA** | **Nominal p value** | **Nearest TSS gene** | **Ensembl ID** |
| --- | --- | --- | --- | --- |
| chr8:48438459-48440458 | 0.200 | 4.53E-05 | NA | ENSG00000269924 |
| chr1:219615485-219617484 | -0.217 | 1.72E-05 | NA | ENSG00000230024 |

Note: uninfected macrophages (CtA: control A), transcription start site (TSS), no available data (NA).

**Figure S1.** Heatmaps of the altered CGIs located at transcription start sites in absence/presence of IL-6.

**
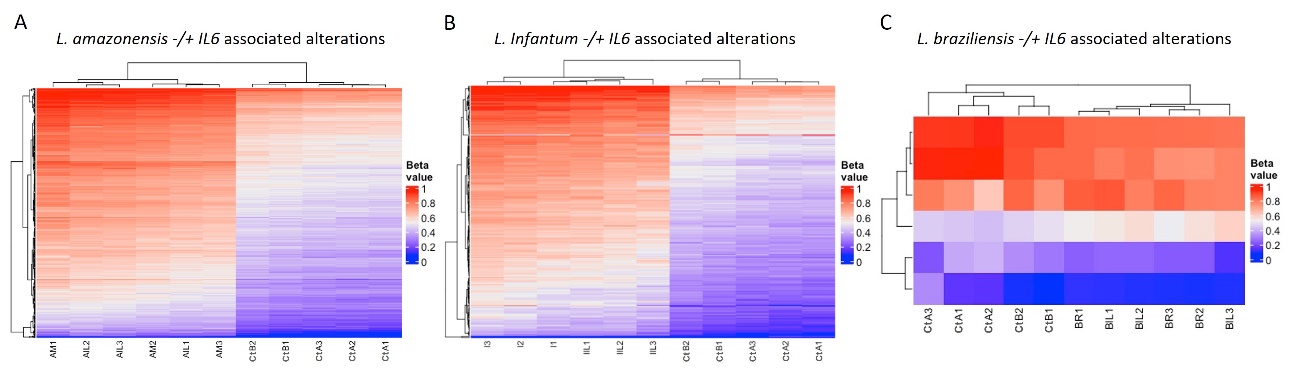
**

UHC analysis based on the beta value of each control and *L. (L.) amazonensis* -/+ IL-6 (A), *L. (L.) infantum* -/+ IL-6 (B) and *L. (V.) braziliensis* -/+ IL-6 (C) infected samples in triplicate. Abbreviations: *L. (L.) amazonensis* + or IL-6 (AIL1, AIL2 and AIL3; AM1, AM2 and AM3); *L. (L.) infantum* + or IL-6 (IIL1, IIL2 and IIL3; I1, I2 and I3); *L. (V.) braziliensis* + or IL-6 (BIL1, BIL2 and BIL3; BR1, BR2 and BR3); control A, uninfected macrophages (CtA1, CtA2 and CtA3) and B, macrophages with heat-killed *Leishmania* (CtB1, CtB2).
